# “Neuro-Fiber Mapping”: An Original Concept of Spinal Cord Neural Network Spatial Targeting Using Live Electrostimulation Mapping to (Re-)Explore the Conus Medullaris Anatomy

**DOI:** 10.3390/jcm12051747

**Published:** 2023-02-22

**Authors:** Philippe Rigoard, Maarten Moens, Lisa Goudman, Tom Le Tutour, Michel Rochette, Jonathan Dany, Mohamed Et Talby, Manuel Roulaud, Rémi Hervochon, Amine Ounajim, Kévin Nivole, Romain David, Maxime Billot

**Affiliations:** 1 PRISMATICS Laboratory (Predictive Research in Spine/Neuromodulation Management and Thoracic Innovation/Cardiac Surgery), Poitiers University Hospital, 86000 Poitiers, France; 2 Department of Neuro-Spine & Neuromodulation, Poitiers University Hospital, 86000 Poitiers, France; 3 PPrime Institute UPR 3346, CNRS, ISAE-ENSMA, University of Poitiers, 86000 Poitiers, France; 4 Department of Neurosurgery, Universitair Ziekenhuis Brussel, Laarbeeklaan 101, 1090 Brussels, Belgium; 5 STIMULUS Consortium (Research and Teaching Neuromodulation uz Brussel), Vrije Universiteit Brussel, Laarbeeklaan 103, 1090 Brussels, Belgium; 6 Department of Radiology, Universitair Ziekenhuis Brussel, Laarbeeklaan 101, 1090 Brussels, Belgium; 7 Research Foundation—Flanders (FWO), 1090 Brussels, Belgium; 8 ANSYS France, 69100 Villeurbanne, France; 9 Department of Oto-Rhino-Laryngologie, Hôpital Pitié-Salpêtrière, 47–83 Boulevard de l’Hôpital, 75013 Paris, France; 10 Department of Physical and Rehabilitation Medicine, Poitiers University Hospital, University of Poitiers, 86000 Poitiers, France

**Keywords:** anatomy, neuroanatomy, conus medullaris, multicolumn spinal cord stimulation (SCS), somatotopy, dorsal columns, computerized electrical modeling, neuro-fiber mapping, super-selective spinal cord stimulation

## Abstract

Spinal cord (SC) anatomy is often assimilated to a morphologically encapsulated neural entity, but its functional anatomy remains only partially understood. We hypothesized that it could be possible to re-explore SC neural networks by performing live electrostimulation mapping, based on “super-selective” spinal cord stimulation (SCS), originally designed as a therapeutical tool to address chronic refractory pain. As a starting point, we initiated a systematic SCS lead programming approach using live electrostimulation mapping on a chronic refractory perineal pain patient, previously implanted with multicolumn SCS at the level of the conus medullaris (T12-L1). It appeared possible to (re-)explore the classical anatomy of the conus medullaris using statistical correlations of paresthesia coverage mappings, resulting from 165 different electrical configurations tested. We highlighted that sacral dermatomes were not only located more medially but also deeper than lumbar dermatomes at the level of the conus medullaris, in contrast with classical anatomical descriptions of SC somatotopical organization. As we were finally able to find a morphofunctional description of “Philippe–Gombault’s triangle” in 19th-century historical textbooks of neuroanatomy, remarkably matching these conclusions, the concept of “neuro-fiber mapping” was introduced.

## 1. Introduction

Spinal cord (SC) anatomy was first described morphologically by pioneers [1,2] before being progressively “animated” with functions by anatomists over the last few decades, based on animal model correlations [3]. The development of immuno-histochemical techniques [4] and access to multiple animal studies [5,6,7] have dramatically deepened our knowledge of this complex entity, where millions of fibers are organized from macroscopic to nanochemical levels [4,8,9,10,11], at the interface of the central and peripheral nervous systems [12,13]. However, functional live anatomy of this neuro-circuitry remains only partially understood [14,15,16]. This could limit our capacity to restore or modulate functions, if needed in the near future [17].

Spinal cord stimulation (SCS) is an electrical technique consisting in implanting lead in the epidural space of the spinal canal, facing the dorsal aspects of the spinal cord, connected to an internal pulse generator (IPG), in order to deliver electrical energy to neural structures, thereby modulating pain perception [18,19]. SCS efficacy has been demonstrated since 1967 [20], and yet, far from the initial gate control theory concerning its main mechanism of action [21], new SCS modalities appear to influence other neural targets and circuits [22,23,24,25] in terms of spatial targeting and temporal resolution. The new generation of available SCS systems now has the ability (i) to shape the electrical field more precisely, given the physical multiplication of columns/contacts at the level of the lead [26,27] and to fragmentate the total amount of energy delivered through multiple sources [28,29], to be more selective on spatial neural targeting, and (ii) to adjust the temporal resolution of the signal, aiming to discriminate intraspinal targeted structures or to guide the information directly to brain integration, via supra-spinal pathways [22,23,24].

**Figure 1:**
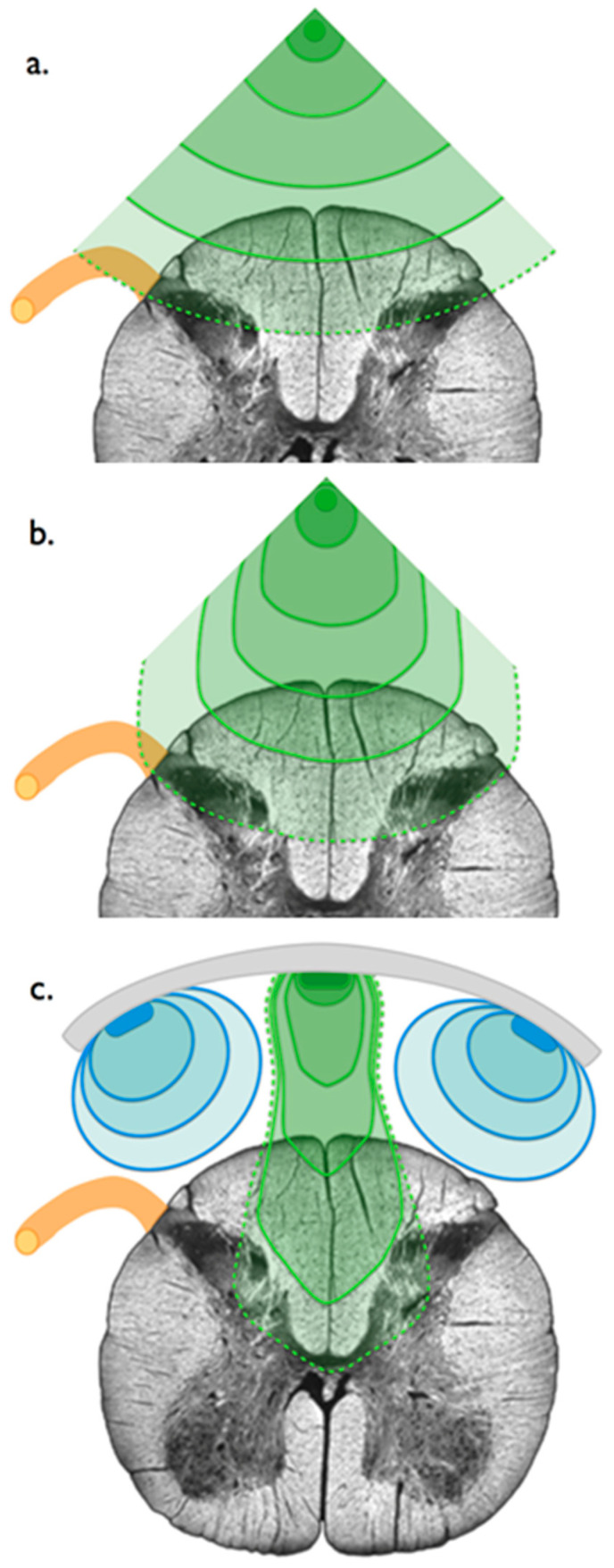
Artistic drawing adapted from Holsheimer et al. [31] representing bipolar, longitudinal tripolar, and transverse tripolar SCS concepts. Schematic views of thoracic spinal cord axial slices showing electrical field simulation within the dorsal columns (DCs) with different programming configurations and corresponding computerized modeling. In orange: right dorsal root; green lines: electrical field stimulation of the DCs; dotted green line: intraspinal but outside of the DC electrical field stimulation generated by an amplitude or pulse with increase. (**a**) Bipolar stimulation. Electrical field stimulation is concentric with recruitment of dorsal root fibers before recruitment of all dorsal column fibers; (**b**) longitudinal tripolar stimulation in a single-column device. According to Holsheimer modeling [31], longitudinal tripolar stimulation allows the electric field to go deeper in the dorsal column fibers before recruitment of dorsal root fibers; (**c**) transversal tripolar stimulation, enabled by independent multicolumn SCS device. By modulating the amplitude of the pulse on both anodes (or more) with various multipolar combinations, it seems possible to focus and steer the electrical field deeper and more laterally within the spinal cord.

Aiming to better understand functional spinal cord neuroanatomy, we hypothesized that using the “super-selectivity” of SCS new modalities, we could re-explore spinal cord neural networks by performing live electrostimulation mapping, providing an opportunity to further combine an anatomical approach to electrical field 3D-computerized modeling, and ultimately to examine the relationship between SCS neural spatial targeting and patient pain coverage, thereby supporting the main mechanism of action (MOA) and clinical efficacy of conventional SCS [24,25]. By “hyperselective coverage”, we mean, if possible, generating exclusive paresthesia over the perineal area (S2, S3, S4, S5 dorsal column sacral projections) without unwanted dorsal root stimulation and/or excessive stimulation of adjacent dermatomal projections, especially regarding the legs (L3, L4, L5, S1 dorsal column projections), also available at this level (Figure 1).

To achieve this goal, we conducted this proof-of-concept study to revisit SC live neuroanatomy, starting at the level of its most caudal region, where the first SC sensitive fibers enter, to the last motor from which fibers exit: the conus medullaris region. With this in mind, we initiated a systematic lead programming approach on a patient implanted with multicolumn SCS at the level of the conus medullaris (T12-L1) to address his complex refractory perineal pain [30]. Our objective was to achieve paresthesia generation and hyper-selective coverage of this patient’s perineal dermatoma and to shape the electrical field much more selectively than with conventional SCS; and to (re-)explore the classical anatomy of the conus medullaris using statistical correlations of paresthesia coverage mappings, issued from the 165 different electrical configurations tested.

## 2. Materials and Methods

### 2.1. Phase 1: Clinical Exploration

The failure of conventional treatment efficacy, the neuropathic component of the complex refractory pelvic pain syndrome, the failure of nerve infiltrations, the contraindication to decompressive surgery, and the efficacy of TENS combined with psychological care led to proposing spinal cord implantation to a 47-year-old man suffering from refractory complex pelvic pain syndrome onset more than for 3 years and combined coccygodynia and bilateral pudendal neuralgia.

#### 2.1.1. Ethical Issues

The patient provided informed consent, and approval was requested and obtained from the Poitiers University Hospital and the French National Committee for Information and Liberty (CNIL, N° 70108724P).

#### 2.1.2. Surgical Implantation

**Figure 2:**
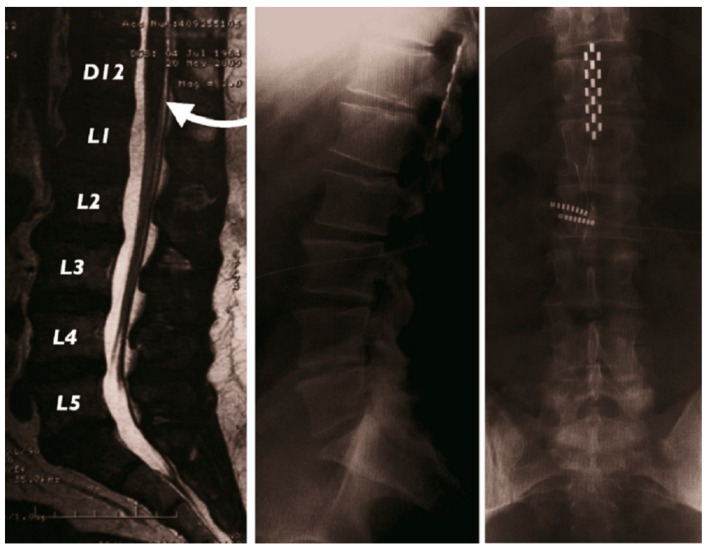
Pre-operative MRI showing the projection of the conus medullaris at T12-L1 vertebral level (on the left) and post-operative X-ray localizing the lead position, at the same vertebral projection (in the middle and on the right)/from Rigoard et al. [32] with permission.

Epidural spinal cord stimulation using 16-contact tripolar surgical lead (Specify^TM^ 5-6-5, Medtronic Inc., Minneapolis, MN, USA) was implanted in a 47-year-old man with a complex refractory pelvic pain syndrome (please find details in ref. [32]). This generation of multicolumn SCS surgical leads, allowing asymmetric stimulation with transverse tripolar configurations (Figure 1), has been designed to allow more targeted stimulation of the dorsal columns without unwanted side effects such as dorsal root (DR) stimulation [33,34] for the treatment of complex chronic pain conditions. The patient was implanted at the level of conus medullaris (T12-L1 vertebral level), identified via preoperative spinal magnetic resonance imaging (MRI) (Figure 2), in order to optimize the positioning of the lead opposite the appropriate vertebrae and allowing for multicolumn stimulation of the painful perineal area. Post-operative X-ray was performed to localize the positioning of the paddle lead.

#### 2.1.3. Electrostimulation Mapping of the Conus Medullaris

Our clinical team worked on statistical algorithms to define specific programs using these tricolumn SCS leads, initially designed to more reliably and specifically target the back dermatomes [34]. A specific exploration of the back, legs, and perineum paresthesia mappings was then performed using series of predetermined multipolar configurations issued from our clinical experience of tricolumn SCS leads in persistent spinal pain syndrome [34].

**Figure 3:**
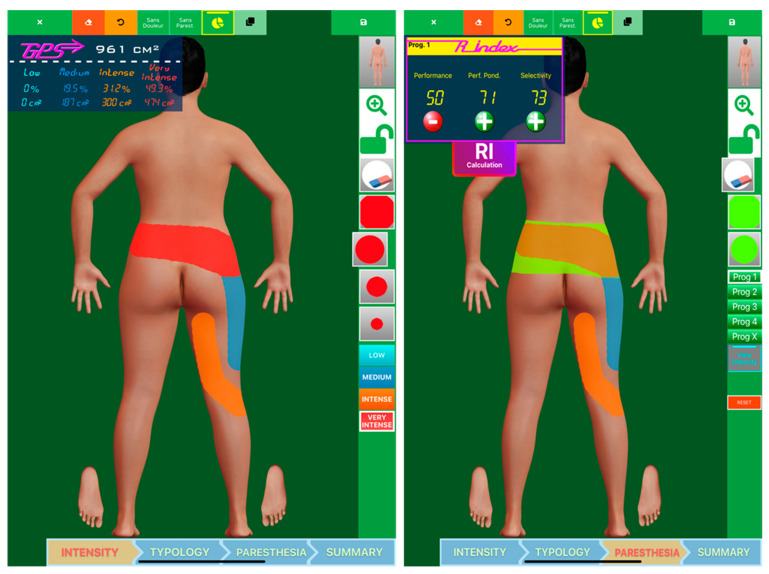
Pain mapping software used to assess pain surface and pain coverage where the patient could draw different painful zones. The pixels in the patient drawing are then converted into cm², using several anatomical landmarks, patient morphology, and morphometry. Four colors are available for patients to represent the different pain intensities. Pain coverage can then be obtained by drawing paresthesia (in green), which is converted into a percentage of pain coverage (performance). With the permission of PRISMATICS Lab.

All in all, 55 different, predefined stimulation patterns were systematically and sequentially tested for this patient, and their respective efficacies in producing paresthesia in the perineal and lower extremity regions were noted on topographic reports via a numerical interface (PRISMap, powered by PRISMATICS, France) (Figure 3, left panel) [29,35,36,37,38]. The patient was explored five times for 30 min to avoid boredom with the iterative electrostimulation mappings, which could have potentially led to a lack of cooperation, for a total testing duration of 2.5 h. The generation of paresthetic sensations (perception threshold) in the legs and/or perineum and/or back, by adjusting the amplitude parameter (in Volts), was recorded on mapping software with tactile interface (PRISMap, powered by PRISMATICS, France) [29,35,36,37,38], along with its “synchronicity” and its topographic predominance depending on the programs used (Figure 3, right panel). The ultimate aim was to ensure increased selectivity in the perineum/leg stimulation ratio.

**Figure 4:**
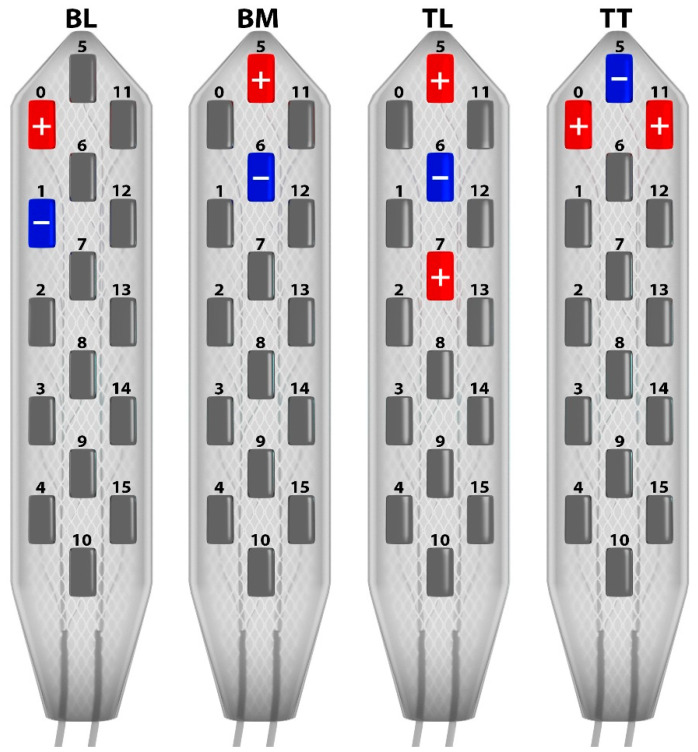
Longitudinal and transverse stimulation patterns. The three groups of stimulation configurations used in this study were BL = bipolar lateral configuration comprising 20 stimulation configurations, BM: bipolar midline configuration comprising 15 stimulation configurations, and TL = tripolar longitudinal configurations comprising 10 stimulation configurations, all with a longitudinal guarded cathode located at the 3rd or 4th rostrocaudal levels of each lead column (3 levels in the left and right columns, 4 levels in the central column). TT = tripolar transverse configurations comprising 10 stimulation configurations, all with a transverse guarded cathode located at the 6 rostrocaudal levels of the lead.

The 55 predetermined stimulation combinations were defined by varying the activation of the 16 contact polarities in order to represent the widest range of multipolar programming possibilities, considering the electrode’s geometry (Figure 4). Out of these 55 “electrical” combinations, 35 were conventional bipolar configurations using one of the three columns; while 20 were bipolar lateral (BL) and 15 were bipolar midline (BM), 10 were tripolar longitudinal (TL) configurations using three adjacent contacts in a linear array (a guarded cathode defined as ‘ + − + ’) on a single stimulation column (also called “monocolumn” in tripolar configurations). The other 10 transverse tripolar (TT) configurations tested used several stimulation columns (also called “multicolumn” configurations), which activated at least three contacts positioned on the medial column of the lead and on at least one of the two lateral columns, using various spatial arrangements of guarded cathodes, as well. Three different pulse durations were analyzed (60, 210, and 450 µs) for a total of 165 configurations (55 × 3 pulse durations). These predefined pulse durations were chosen not only because we were limited by the patient’s cooperation, but also by the fact that our previous experience [34] had shown that the pulse width duration did not have as much of an influence on the initial paresthesia coverage as in extending the field of coverage during a second phase of programming. The whole range of frequencies was also tested on configurations able to generate paresthesia in the perineum area and analyzed.

#### 2.1.4. Data Analysis

A total of 55 configurations were tested at variable amplitudes, frequencies, and pulse durations. Combining all intensities and pulse durations, the data analysis was carried out based on 165 tests with 60 configurations for conventional bipolar lateral (BL), 45 for conventional bipolar midline (BM), 30 for tripolar longitudinal (TL), and 30 for tripolar transverse (TT). The main outcome measure was the number of positive responses assessed with bilateral coverage of paresthesia in the perineum and lower extremity regions for each of the 165 tests for the 4 configurations (i.e., BL, BM, TL, TT). The patient was asked to specify whether or not he could feel the paresthesia and its location.

Meticulous monitoring of bilateral/anterior and posterior perineum paresthesia perception was analyzed in the perineal region during the trial. This number was calculated for each of the three configuration groups, and then for all of the multicolumn configurations.

## 3. Results

### 3.1. Programming Sessions

We generated paresthesia in the legs and the perineum by programming the multicolumn lead in this patient at the level of the conus medullaris. We started the programming session by using bipolar configurations on the lateral (BL) then medial (BM) column, followed by longitudinal tripoles (TL), and we finally explored the potential of the lead with transverse tripolar (TT) configurations. Variations in pulse width successfully provided wider coverage at times, resulting in disturbing paresthesia over the painful area, which was regarded as an adverse effect. By increasing amplitude stimulation above the paresthesia perception threshold up to the comfort threshold, we observed in some configuration uncomfortable sensations in the legs before successful targeting of the buttock.

### 3.2. Monocolumn Combinations

**Figure 5:**
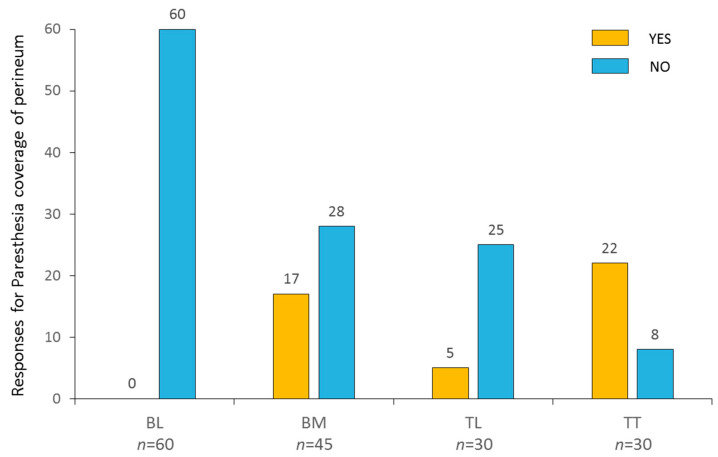
Mapping/programs/statistics correlation. BL: bipolar midline; BM: bipolar midline; TL: tripolar longitudinal configuration; TT: tripolar transverse configuration.

**Figure 6:**
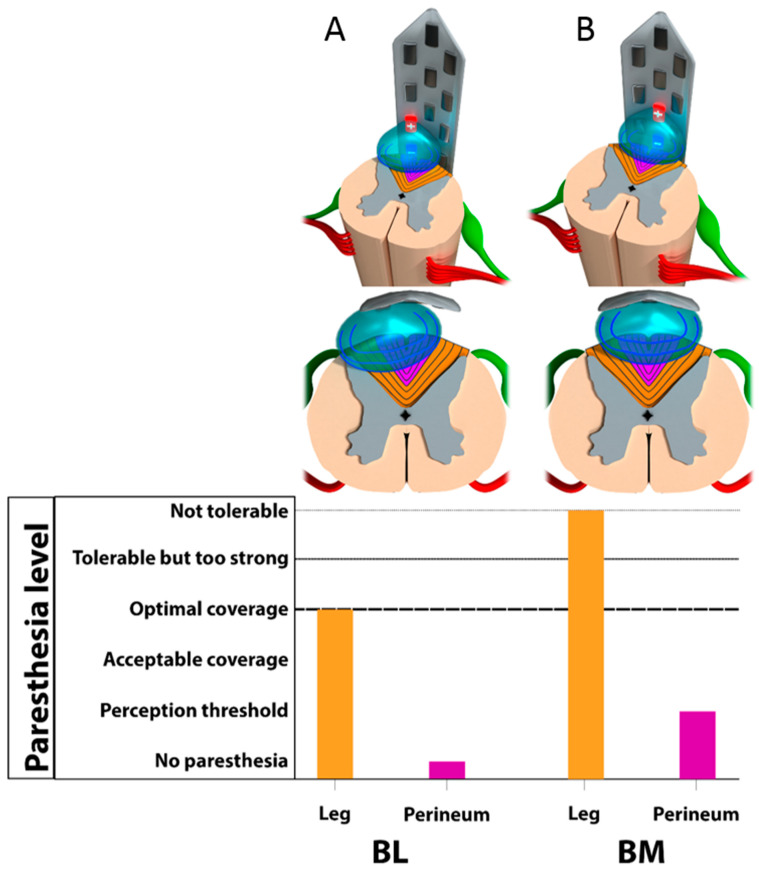

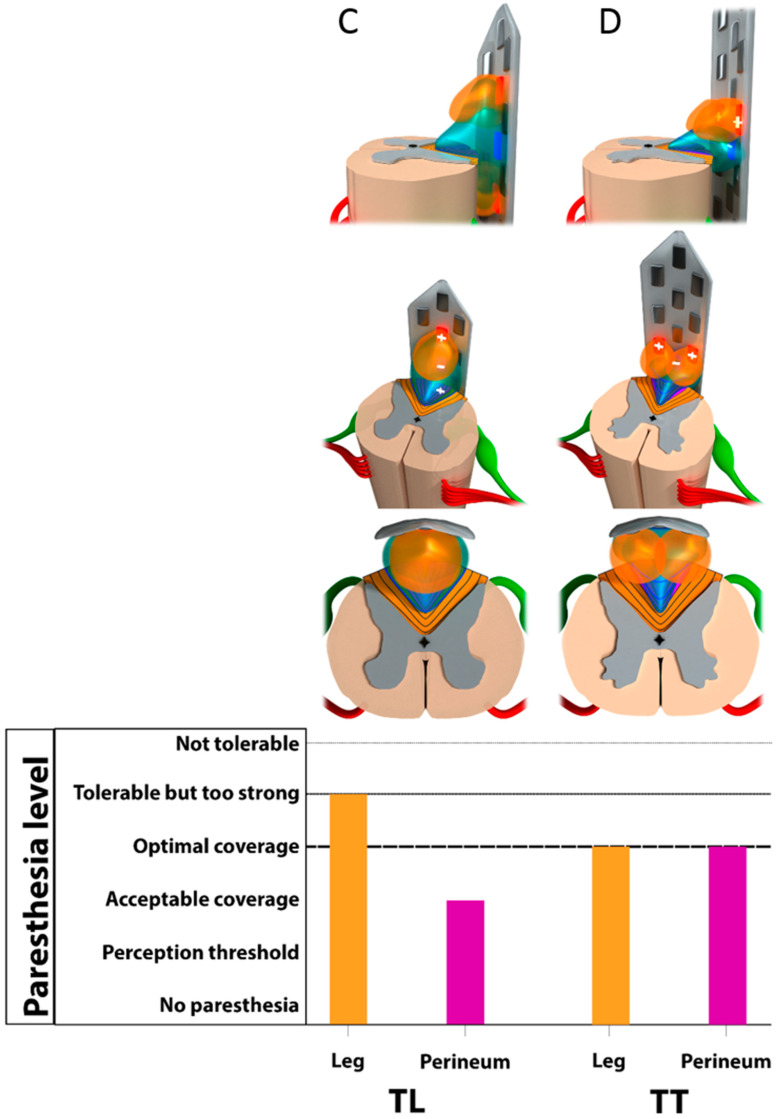
Anatomical representation of Philippe–Gombault’s triangle at the conus medullaris. L: leg projections corresponding to lumbar dermatomes; P: perineum projections corresponding to sacral dermatomes. Neuro-fiber mapping (NFM) of the conus medullaris. (**A**) Bipolar lateral (BL) configuration; (**B**) BM: bipolar midline configuration; (**C**) tripolar longitudinal (TL) configuration; (**D**) tripolar transverse (TT) configuration.

*Bipolar lateral configurations*. None of the 60 BL combinations tested generated paresthesia in the perineum area, as it was localized on the lateral column (Figure 5). In this case, stimulation was always felt in lower limb dermatome, from L3 to L5 (Figure 6).

*Bipolar midline configurations*. BM combinations (15 × 3) achieved paresthesia in the S1 dermatome for 31/45, and in the perineum in S2 to S5 dermatomes for 17/45 (Figure 5). More specifically, the 210 µs pulse width was mainly used on these successful combinations (10/17 perineum coverage). However, although perineal pain was covered, the paresthesia felt by the patient in the lower extremities was too strong to allow us to use these settings in the long term (Figure 6).

*Tripolar longitudinal configurations*. In all, 5 out of the 30 TL configurations (10 × 3) achieved bilateral paresthesia in the perineum, which was always observed for midline combinations (Figure 5). It was possible to cover the S2 dermatome with only two of the lateral combinations. In all of these cases, however, as the perineal pain was covered, strong but intolerable paresthesia was systematically felt by the patient in the lower extremities (Figure 6).

In summary, for monocolumn configurations (A + B), we observed possible coverage of the targeted area but lack of selectivity, compromising its use on daily practice.

### 3.3. Multicolumn Combinations

*Tripolar transverse configurations*. TT provided the best results in terms of paresthesia coverage and selectivity (22/30 perineum coverage), by using multicolumn programs (Figure 5). Out of the 30 TT combinations (10 × 3), 20 generated bilateral paresthesia in S3, S4, and/or S5 dermatomes. The S2 dermatome was covered for 2 out of 30. The S4 and S5 dermatomes were the most frequently covered, with 10 out of 30 for S4 and 19 out of 30 for S5. In two cases of TT combinations, isolated paresthesia was obtained exclusively over the perineum with no irradiation in the lower limb, even in the S1 dermatome (Figure 6).

All in all, out of the 165 combinations tested, 44 (0/60 lateral bipolar; 17/45 midline bipolar; 5/30 longitudinal tripolar and 22/30 transverse tripolar combinations) allowed adequate coverage of the painful perineal area (i.e., S2, S3, S4, and/or S5). Ultimately, the patient was stimulated with a combination of two of these programs with 100% coverage of the painful area. No adverse effects (uncomfortable DR stimulation) were observed with the final settings.

## 4. Discussion

This proof-of-concept project arose from a clinical perspective: while SCS is a well-established therapy to address chronic refractory pain, its MOA remains unclear. When applying SCS in front of patient dorsal columns, it appears impossible to precisely determine which microscopic neural structures among billions of fibers are electrically targeted by SCS. The reason for this is quite simple: SC functional neuroanatomy has been described in textbooks with few and limited functional extrapolations based on clinical human anatomy.

Therefore, we took the opportunity to start by enrolling a patient implanted with multicolumn SCS for refractory perineal pain and to use new SCS spatial targeting modalities, the objective being to explore his SC neural circuitry with live electrostimulation mapping. While multicolumn epidural SCS leads are usually implanted at the level of thoracic spinal cord dorsal columns, T8-T10, their implantation at a lower level such as the conus medullaris has only recently been reported [32,39].

Our study comprises a systematic programming approach (Phase 1), which will be further correlated to a computerized modeling (Phase 2) and will lead us to “electronically” dissect the somatotopical organization of the dorsal funiculi at the level of the conus medullaris, by means of live anatomo-electrophysiological comparisons. We considered these initial steps of neural re-exploration through live electrostimulation mapping as a premise of the “neuro-fiber mapping” (NFM) concept.

We hypothesized that we could (a) generate a highly focused neural stimulation field in the dorsal funiculi using multicolumn programming combinations and (b) steer the current within the dorsal columns in order to selectively depolarize deeper and/or lateralized layers of ascending afferent fibers. These technological capabilities characterizing a new type of SCS, which could be called a “super-selective SCS” (SSSCS), may be considered “a research vehicle” to (re-)explore neural structures in implanted SCS refractory pain patients.

Through a gradual approach, we first presented a traditional view of the conus medullaris neuroanatomy. In the second part attempting to extrapolate novel correlations from our electrostimulation mapping combination data, we shall put our research in perspective with original neuroanatomical considerations from pioneering works of the 19th century. In the final part, we stress the strengths and limitations of this study, which would require research on a larger scale to clinically validate the concept of NFM using SSSCS.

### 4.1. Part I—Classical Anatomy and Somatotopical Organization of the Conus Medullaris

#### 4.1.1. Neuroanatomical Textbook Data

**Figure 7:**
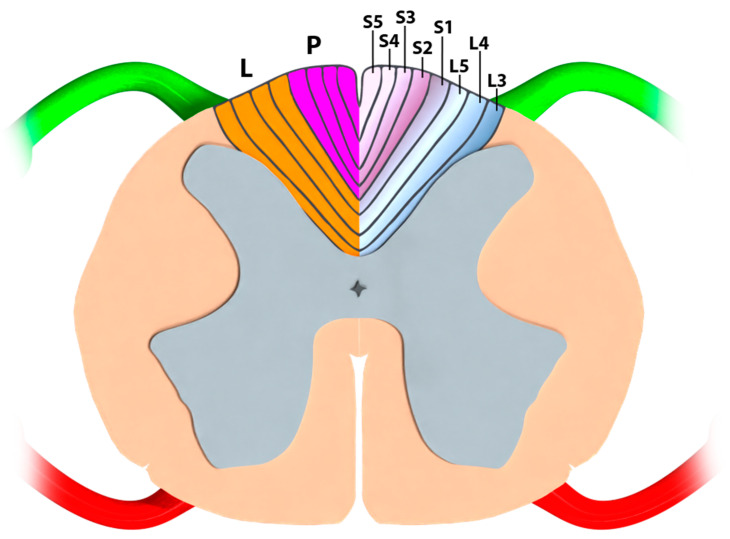
An artistic view illustrating classical dorsal column somatotopy at the conus medullaris level. L: leg projections corresponding to lumbar dermatomes; P: perineum projections corresponding to sacral dermatomes.

The myelinated dorsal root fibers of the dorsal columns, which carry tactile and proprioceptive information, are somatotopically organized fibers [3] from the sacral and lumbar roots that ascend medially in the fasciculus gracilis [4]. The classic somatotopic description of the birth of the fasciculus gracilis at the level of the conus medullaris shows that the first and lowest afferents coming into the dorsal columns (S5, then S4, S3, etc.) pile from the midline to the lateral dorsal white matter area [40] and that lumbar dermatomes (i.e., leg projections) are more lateral than sacral dermatomes (i.e., perineum projections) (Figure 7). However, it is very difficult to find precise data in the majority of neuroanatomy textbooks to deepen our understanding of the functional organization and the microanatomy of dorsal funiculi at the level of the conus medullaris [2,3,4,8,9,11]. Moreover, there are few available data regarding the precise anatomical or electrophysiological relationships between the somatotopical organization of the dorsal funiculi and the levels of spinal cord.

#### 4.1.2. Anatomo-Electrophysiological Correlations Regarding a Clinical Blackbox

Historically, some electrophysiological monitoring procedures have been described at the level of the conus medullaris. They used sacral nerve root stimulation for micturition in patients with spinal injuries [4,41]. However, this testing using peripheral nerve monitoring was carried out under general anesthesia, without the cooperation of the patient, and concerned direct stimulation of the ventral roots exploring visceral functions (i.e., bladder micturition and anal striate sphincter constriction) without elective exploration of the somatosensory tracts of the peripheral nervous system [42]. No electrophysiological correlation to the morphological anatomy and somatotopy of the conus medullaris has yet been precisely evidenced.

The main question is the following: Why do we observe, especially using traditional midline SCS lead programming, that paresthesia generation (i.e., spatial neural targeting) is easier to perform and remains predominant in the legs rather than the perineum, even though the sacral dermatomes are characterized in classical somatotopy and electrophysiological models as the most median and superficial?

Consequently, in conventional practice, it is almost impossible for an implanter using monocolumn spinal cord stimulation (SCS) leads to reliably predict the optimal target for neuromodulation that would specifically address the sacral dermatomes. In addition, lack of selectivity represents one of the major limitations of SCS, since it appears extremely difficult to selectively depolarize the sacral dermatomes at the conus medullaris level. In fact, lumbar dermatomes are distributed more laterally at this level, and we expected to cover them first with lateralized fields of stimulation generated by single bipolar lateral-column activations. This result would fit perfectly with the classical somatotopic description [3,43] of the initial part of the dorsal columns at the conus medullaris (Figure 7).

Surprisingly, using bipolar midline monocolumn programs, paresthesia was almost systematically generated in the legs (lumbar dermatomes) before the perineum (sacral dermatomes) (Figure 6). In contrast to the previous findings, this does not match with what we would expect from the classical description of somatotopical organization and computerized modeling.

Given these unexpected electrophysiological data, the most reasonable and logical hypothesis would be that, in opposition to classical anatomy, perineal projections should be deeper than leg projections at this stimulated level. While this would explain why we depolarize legs before perineum, it cannot explain why we would find any leg fiber, at the extreme distal end of the spinal cord, without having depolarized any sacral fiber earlier (sacral fibers, as characterized in Section 4.1, being the first and the most distal fibers available at the very end of the conus).

By using longitudinal tripoles, according to Holsheimer’s team theory [33], it appears possible to steer the current deeper in the dorsal columns. Using our NFM technique, through neuro-mapping involving several longitudinal tripolar programs, we observed increasing recruitment of sacral dermatomes (and also a slight increase in perineum/leg coverage ratio as a consequence), even though it was almost impossible not to strongly stimulate the lumbar dermatomes associated with these programs (Figure 6). We thereby confirmed our hypothesis that sacral fibers are “hiding” and that to shape the electrical field, they must be depolarized.

Our last transversal neuro-mapping revealed that 20 out of the 30 transverse tripolar programs generated paresthesia predominantly in the perineum and produced more selective sacral dermatomes coverage, by hyperpolarizing the laterality, thereby ruling out collateral stimulation of the lumbar dermatome projections in the dorsal columns (Figure 5 and Figure 6). We observed that the sacral/lumbar dermatome recruitment ratio switched in favor of the perineum area, allowing much larger coverage of the perineal pain and much more “tolerable” coverage of the pain in the lower extremities.

Finally, we can conclude that (1) some of our data issued from NFM of the conus medullaris, are in accordance with the classical somatotopy description of the dorsal columns (i.e., leg dermatomes are more lateral than perineum dermatomes at the conus), but (2) other observations were unexpected and do not fit with the recent literature (i.e., leg dermatomes seem to be more superficial than perineum dermatomes at the conus).

#### 4.1.3. Historical Revisitation of the Morphological Anatomy of the Dorsal Funiculi at the Conus Medullaris

To document this gap between classical morphological anatomy and our NFM findings, we researched anatomical textbooks of the past centuries to ascertain alternative anatomical structures or neural networks discovered during the “golden age” of anatomy that would support our electrostimulation mapping of the conus medullaris.

We performed a bibliographical review of dorsal funiculi functional anatomy from the late 19th century, disclosing a little-known interneuronal network juxtaposed to the midline and connecting overlying lumbar dermatomes to sacral ones at the conus level. This fascinating entity was described as an “intramedullar commissural endogenous fibers network”.

##### Notion of Endogenous Fibers and Intraspinal Commissural Pathways

Cells of the various superimposed layers in the spinal cord come into reciprocal contact [44]; neuron associations or intercalary neurons infiltrate in a staggered manner over the entire height of the gray axis [45]. The dorsal column contains root nerve fibers from the spinal ganglia that intermingle with the fibers of the intercalary neurons, which come from cells of gray matter [46]. As the cells from which they emanate are contained inside the spinal cord, the association fibers of the dorsal column are called endogenous fibers, as opposed to root fibers arising outside the spinal cord, which are referred to as exogenous fibers [45].

The posterior root fibers are exogenous and ascending. Impairment of these fibers, in Tabes pathology, explains fulgurant pain and anesthesia; their destruction induces tactile anesthesia. The endogenous fibers are short, medium, and long association fibers. Some are ascending, others descending [47]. These endogenous fibers, preserved in Tabes pathology, are affected in diseases of the dorsal columns of endogenous origin (general paralysis, pellagra, ergotism) [46]. Study of these degenerations has clarified their position in the dorsal column over bundles of exogenous root fibers.

##### Original Anatomical Description of the Inter-Commissural Endogenous Fiber Network by Philippe

In 1897, a pathologist named Claudien Philippe presented data in his thesis regarding ascending and descending endogenous fibers [48]. (a) Ascending endogenous fibers: their existence is proved by the persistence of an intact area behind the posterior commissure in the degeneration pathology resulting from a lesion of the cauda equina and, secondly, by altering the same area, above a destructive intramedullary source that remains intact below. This area in the whole height of the posterior column is called the cornu-commissural area of Marie. It has the sectional shape of a small croissant packed against the antero-internal part of the dorsal horn near the commissure and in front of the cornu-radicular area that it seems to extend. (b) Descending endogenous fibers: their topography has been established by studying the areas of degeneration that can be observed below a limited transverse lesion of the spinal cord; the corresponding areas are observed in levels only above the lesion.

**Figure 8:**
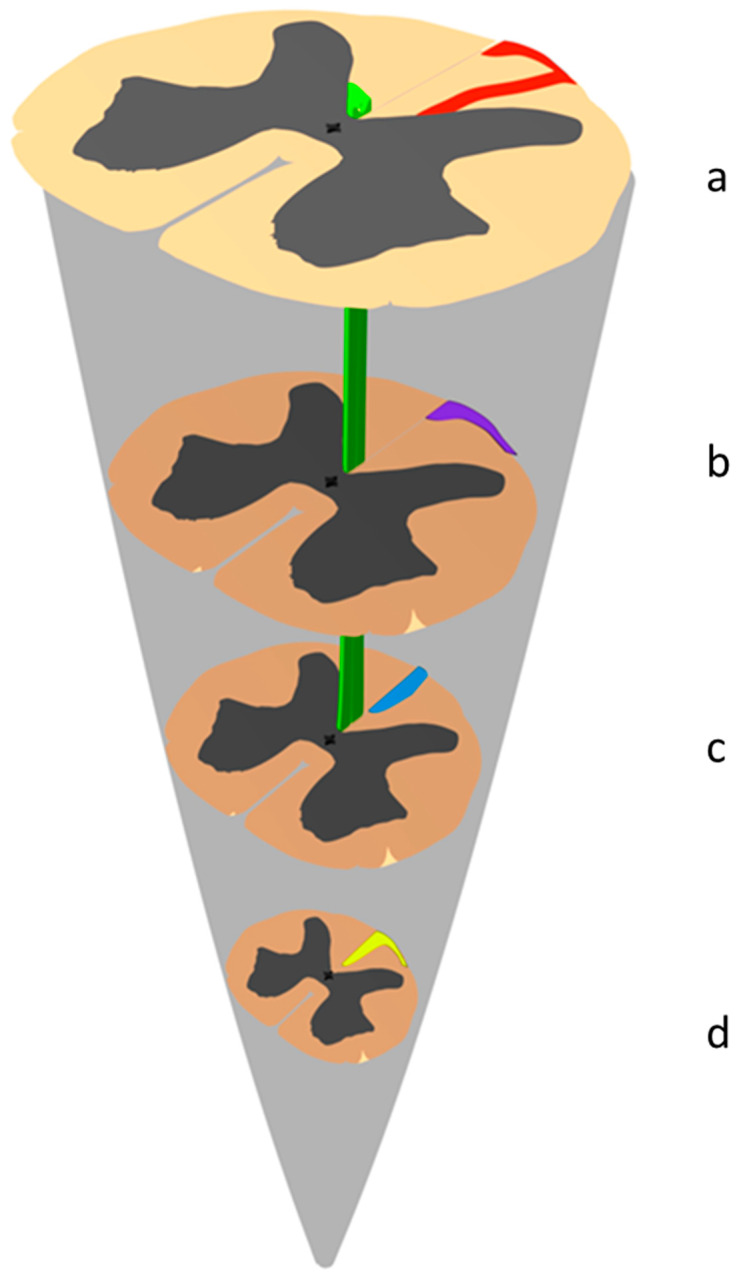
Fiber system and endogenous descending posterior commissure pathways (original graphic conception by JP Giot and P Rigoard, drawn from Philippe [15]). (**a**) In the cervical and upper dorsal spinal cord, fibers of this bundle appear as a strip included in the columns of Burdach. Because of their shape, they are called the comma tract of Schultze (in red); (**b**) in the upper lumbar and dorsal spinal cord, the previous bundle deforms and flattens into a strip which extends at the periphery of the dorsal column and is termed the peripheral posteromedial strip of Soucques and Marinesco (referred to, by Marie, as the posteromedial area of Flechsig in purple); (**c**) in the lower lumbar spinal cord, this bundle takes on the form of a biconvex lens in contact with the median sulcus; its anterior end does not reach the commissure, while its posterior end remains on the periphery. This bundle is called the oval area of Flechsig (in blue); Marie’s cornu-commisural zone in green in (**a**–**d**). In the sacral spinal cord and into the conus medullaris, this bundle occupies a small triangle attached to the median sulcus; the base of this triangle corresponds to the surface of the spinal cord, and its tip to the posterior commissure. This is the median triangular bundle of Gombault and Philippe (in yellow).

These fibers are grouped into a single bundle, composed of descending root fibers, whose position within the exogenous fibers of the dorsal column varies depending on the selected region of the spinal cord under consideration (Figure 8) [49,50]. In the cervical and upper dorsal spinal cord, fibers of this bundle appear as a strip included in the columns of Burdach called the comma tract of Schultze related to their shape (Figure 8a). In the upper lumbar spinal cord, this bundle is called the oval area of Flechsig (Figure 8b). In the lower lumbar and dorsal spinal cord, the previous bundle is termed the posteromedial area of Flechsig (Figure 8c). In the sacral spinal cord and into the conus medullaris, this bundle occupies a small triangle attached to the median sulcus called the median triangular bundle of Gombault and Philippe (Figure 8d).

The four areas listed above and located in the various segments of the spinal cord respond to a single fiber system that Gombault and Philippe characterized as endogenous long fibers, descending to the conus medullaris [49,50]. This descending endogenous fiber bundle gradually moves inwards as it approaches the conus medullaris. Its displacement is comparable, but inverse, to that experienced by ascending root fibers, which are closer to the median sulcus the further they rise.

In his 1898 treatise, “Systematic nervous affections and theory of neurons,” Gerest stated [45]: “*The continuity of the bundle as described above in its various stages is not yet accepted by all authors. It is clearly stated in the thesis of Philippe, but research by Hoche and Dufour led them to conclude that this bundle exists in two fiber systems: one short (forming the comma of Schultze);the other long, extending into the peripheral strip, the center oval and the median triangle. Even though this point still under discussion, it has nevertheless been established that in the dorsal columns, there are ascending and descending fibers, stemming from the dorsal horn and ending there after a shorter or longer displacement within the root fibers. These fibers are the cylindraxis serving as a sort of bridge between cells at different stages of the dorsal horn, each one of them assuming a role in sensory conduction. It was logical to link study of sensory pathway to study of intercalary association neurons between sensory neurons.*”

Lastly, the cornu-commissural area, the center oval of Flechsig, the triangle of Gombault and Philippe, and the comma of Schultze consist of exogenous and endogenous fibers [51]. These four areas belong, at different stages, to the same descending endogenous fiber bundle (Figure 6). These cells send commissural extensions of three types [44]: one in the same half of the spinal cord (ipsilateral or tautomers), others on the opposite side (heteromers or controlateral), and still others going by bifurcation in the same half and on the opposite side (hecateromers of Van Gehuchten) [52].

##### Description of the Triangle of Philippe and Gombault

**Figure 9:**
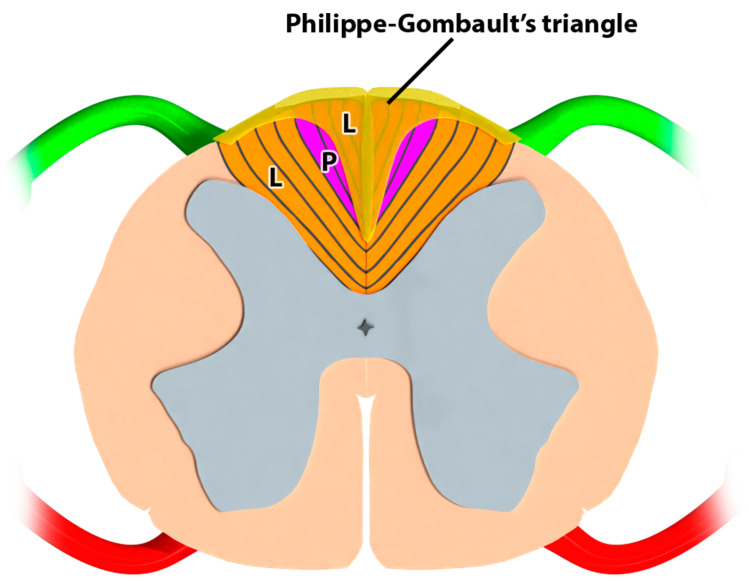
Anatomical representation of Philippe–Gombault’s triangle at the conus medullaris. L: leg projections corresponding to lumbar dermatomes; P: perineum projections corresponding to sacral dermatomes.

The triangle of Philippe and Gombault occupies the periphery of the spinal cord at the conus terminalis (Figure 9) and is mounted on the rear end of the medial septum; this is a very narrow bundle, composed of only a few fibers. In cases of spinal cord injury [46], this triangle degenerates in both directions: from top to bottom, following an inferior dorsal or lumbar transversal spinal cord lesion; and from bottom to top, following a lesion of the sacral roots. This indicates that it contains fibers descending from the lumbar metamers.

#### 4.1.4. Historical Anatomical and Clinical Correlations

Philippe [48] also proved that the initial Tabes reaches the posterior radicular system from the root to the corresponding column in the spinal cord (with an election for medium fibers of Singer and Muenzer) [52] and argued that advanced Tabes was characterized by the destruction of descending endogenous areas.

The importance that Philippe assigned to the destruction of endogenous area is explained by his definition of endogenous zones: “areas that are actually mixed, because they contain both radicular and spinal cord fibers: the mixed composition of the comma of Schultze, of the oval center of Flechsig, of the triangle of Gombault and Philippe, of the cornu-commissural area is something absolutely proven”.

Garcia described the same type of degeneration as part of tuberculosis spinal cord injury: “First, we see that the degeneration appears at the first lumbar root, where it occupies a triangular area representing about half of the posterior bundle. In a section through the cervical region, advanced degeneration can be observed and limited to the columns of Goll. The commissural fibers are intact as well as those in the oval center of Flechsig, and also in the triangle of Gombault and Philippe” [53].

### 4.2. Part III-Study Strengths and Limitations

#### 4.2.1. An Alternative Approach Based on Neuron Electrical Properties and 3D-Computerized Modeling

We based our hypothesis on an anatomical rationale and a modified somatotopical organization within the dorsal funiculi to support our concept of NFM. However, we must admit that, for the moment, we may have missed out on some information due to a lack of 3D-SC-computerized modeling, to confirm/refute our hypothesis, as another face of the prism. This alternative electrical approach should bear in mind that lumbar fibers and sacral fibers could have different morphofunctional characteristics (i.e., myelination, size distribution, etc. and different electrical properties). This could explain a variation of sensitivity generating a gradual involvement of two different neuronal populations influenced by the same electrical field, without any specific anatomical somatotopy arrangement. The objectivation of a lack of any difference between epicritic lumbar and sacral fibers’ electrical properties should be a prerequisite for further studies to validate/question the concept of NFM.

#### 4.2.2. Historical Limitations

Our investigations were limited by the paucity of historical material, and conclusions and extrapolations should be drawn with caution.

In summary, NFM of the conus medullaris enabled us to confirm that leg dermatomes are more lateral than perineum dermatomes at the conus level, as illustrated in the classical somatotopy of the dorsal columns. However, in contrast to the recent literature, we found that leg dermatomes seem to be more superficial than perineum dermatomes at the conus level, resulting in generation of paresthesia in the legs before the perineum. This finding seems to correlate well with the morphological 19th century characterization of Philippe–Gombault’s triangle. Although our study has certain limitations (particularly a missing alternative bioelectrical approach, which could highlight variations in susceptibility to the electrical field, from lumbar and sacral fibers), we believe that electrostimulation correlations, coupled with computerized modeling, could help to explore spinal cord networks by means of neuro-fiber mapping (NFM). The ultimate goal of NFM would be to use it as a clinical tool to provide more selective paresthesia generation and greater coverage of painful areas, thanks to technical SCS evolution toward super-selective SCS. This approach might be optimized by using awake anesthesia in order to test programming stimulation intraoperatively assessed with our mapping tool specifically dedicated to determination of paresthesia coverage related to initial pain area [54].

## 5. Conclusions

Spinal cord implantation of a new generation of multicolumn surgical lead, at the level of the conus medullaris, revealed a correlation between our electrostimulation mapping of the dorsal columns and a morphological 19th-century description of Philippe–Gombault’s triangle. At the level of the conus medullaris, leg projections corresponding to lumbar dermatoma are not only more lateral than perineum projection corresponding to perineal dermatoma but also more superficially, and regrouped as a bundle called Philippe–Gombault’s triangle. The concept of re-exploring spinal cord networks by neuro-fiber mapping is introduced and seems to be possible with a new generation of SCS devices able to provide super-selective spinal cord stimulation. This could delineate a cornerstone in the use of neuromodulation, not only as a tool to provide more selectivity on paresthesia generation and coverage of painful territories, but also as an opportunity to highlight the importance of constant revisitation of classical neuroanatomy, maintaining this discipline as a “live” and dynamic science. Further explorations should benefit from additional correlations arising from the next generation of 3D-computerized anatomical and bioelectrical SCS models.

## Data Availability

Not applicable.
